# Glucose Transporters in Diabetic Kidney Disease—Friends or Foes?

**DOI:** 10.3389/fendo.2018.00155

**Published:** 2018-04-09

**Authors:** Anita A. Wasik, Sanna Lehtonen

**Affiliations:** Department of Pathology, University of Helsinki, Helsinki, Finland

**Keywords:** diabetic kidney disease, glucose transporters, insulin resistance, insulin signaling, podocyte, type 2 diabetes

## Abstract

Diabetic kidney disease (DKD) is a major microvascular complication of diabetes and a common cause of end-stage renal disease worldwide. DKD manifests as an increased urinary protein excretion (albuminuria). Multiple studies have shown that insulin resistance correlates with the development of albuminuria in non-diabetic and diabetic patients. There is also accumulating evidence that glomerular epithelial cells or podocytes are insulin sensitive and that insulin signaling in podocytes is essential for maintaining normal kidney function. At the cellular level, the mechanisms leading to the development of insulin resistance include mutations in the insulin receptor gene, impairments in the phosphoinositide 3-kinase (PI3K)/AKT signaling pathway, or perturbations in the trafficking of glucose transporters (GLUTs), which mediate the uptake of glucose into cells. Podocytes express several GLUTs, including GLUT1, GLUT2, GLUT3, GLUT4, and GLUT8. Of these, the most studied ones are GLUT1 and GLUT4, both shown to be insulin responsive in podocytes. In the basal state, GLUT4 is preferentially located in perinuclear and cytosolic vesicular structures and to a lesser extent at the plasma membrane. After insulin stimulation, GLUT4 is sorted into GLUT4-containing vesicles (GCVs) that translocate to the plasma membrane. GCV trafficking consists of several steps, including approaching of the GCVs to the plasma membrane, tethering, and docking, after which the lipid bilayers of the GCVs and the plasma membrane fuse, delivering GLUT4 to the cell surface for glucose uptake into the cell. Studies have revealed novel molecular regulators of the GLUT trafficking in podocytes and unraveled unexpected roles for GLUT1 and GLUT4 in the development of DKD, summarized in this review. These findings pave the way for better understanding of the mechanistic pathways associated with the development and progression of DKD and aid in the development of new treatments for this devastating disease.

## Introduction

Diabetic kidney disease (DKD) is the serious complication of diabetes. Clinically, DKD manifests as progressive albuminuria and gradual decline in estimated glomerular filtration rate. The cumulative incidence of DKD in patients with type 1 diabetes (T1DM) is 20–40% after 20–25 years of diabetes (1). Type 2 diabetes (T2DM) may remain undiagnosed for several years after onset of the disease, and thus patients may have DKD already at the time of diagnosis. The prevalence of low-level albuminuria in patients with T2DM has been reported to be 24.9% 10 years after diagnosis (2). DKD due to either T1DM or T2DM is the leading cause of end-stage renal disease.

**Figure 1:**
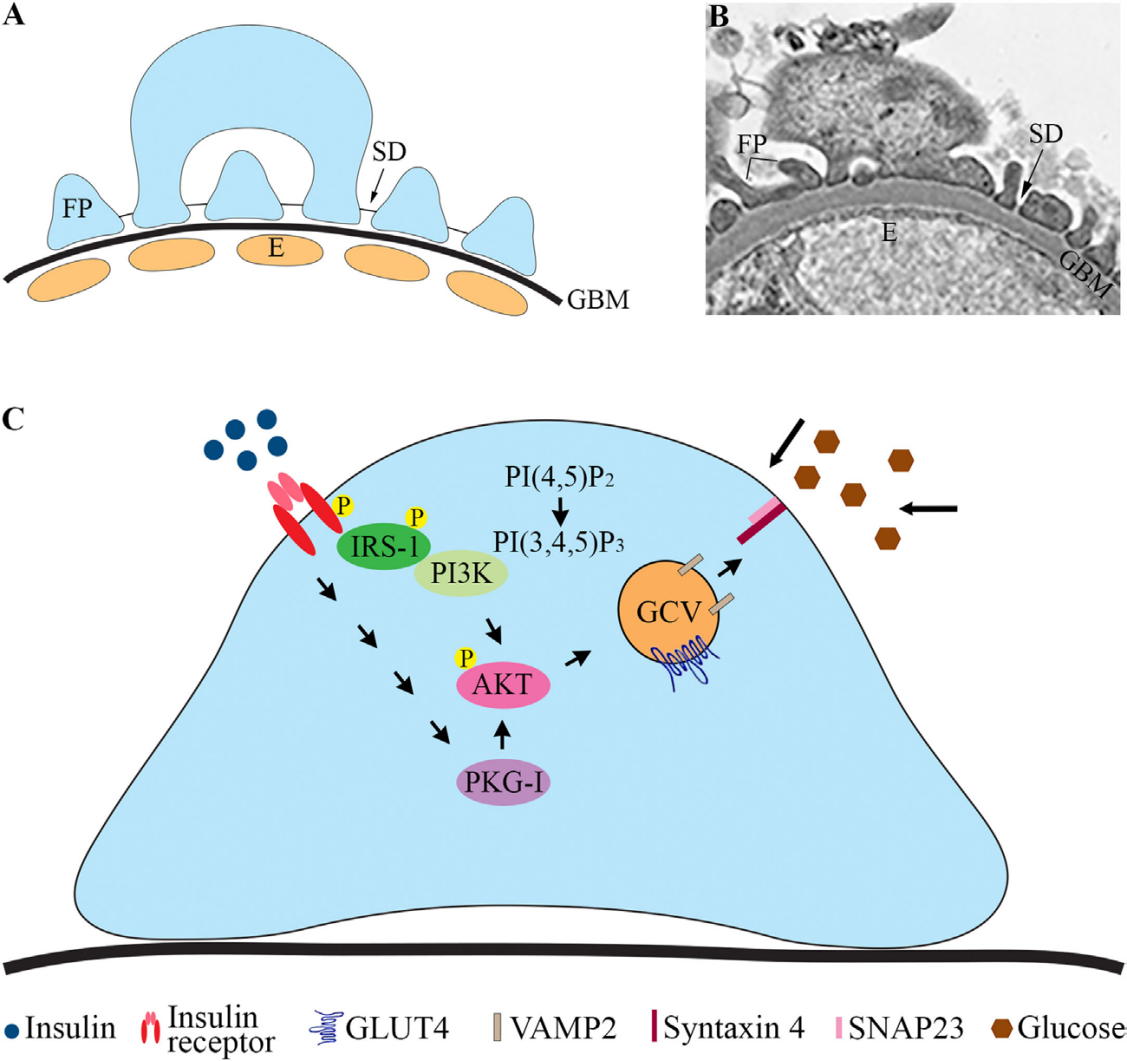
Glomerular filtration barrier (GFB) and schematic cartoon of the insulin signaling pathway in podocytes. **(A)** Schematic structure of GFB. **(B)** Electron microscopic image of the GFB in mouse. **(C)** Insulin activates the insulin receptor tyrosine kinase, which recruits and phosphorylates different substrate adaptors, such as the IRS family proteins. Tyrosine-phosphorylated IRS-1 binds PI3K. The catalytic subunit of PI3K phosphorylates phosphatidylinositol (4,5)-bisphosphate (PIP2) leading to the formation of phosphatidylinositol (3,4,5)-trisphosphate (PIP3). PI3K is also linked to the activation of the protein kinase Akt (protein kinase B), and further downstream to the translocation of GCVs to the plasma membrane. Also, activation of PKG-I leads to enhanced phosphorylation of AKT and increases glucose uptake. E, endothelial cell; FP, foot process; GBM, glomerular basement membrane; GCV, GLUT4-containing vesicle; IRS-1, insulin receptor substrate-1; P, phosphorylation; PI3K, phosphoinositide 3-kinase; PKG-I, cGMP-dependent protein kinase G type I.

The pathophysiological mechanisms leading to the development of DKD remain incompletely understood. Genetic factors, inflammation, and metabolic disturbances are known to be involved [reviewed in Ref. (1)]. Hyperglycemia plays a central role, and also insulin resistance is a risk factor for DKD and contributes to the development of the disease (3). This is supported by studies showing that insulin resistance correlates with microalbuminuria in diabetic (4–7) and non-diabetic subjects (8). Albuminuria develops when the glomerular filtration barrier (GFB), consisting of the fenestrated endothelial cells, the glomerular basement membrane (GBM), and the glomerular epithelial cells or podocytes (Figures 1A,B), is disturbed. Podocytes are polarized, highly specialized, and terminally differentiated cells. The foot processes of neighboring podocytes interact with specialized cell to cell junctions called slit diaphragms (SD) located between the basal and apical domains of the foot processes (Figures 1A,B). Podocyte injury has been suggested to have a pivotal role in the pathogenesis of DKD (9). During podocyte injury, podocytes efface (flatten) and detach from the GBM, and the frequency of SDs is reduced.

Interestingly, podocytes are insulin sensitive (10) and have been shown to develop insulin resistance in animal models of diabetes (11). Concomitantly, insulin resistance has been proposed as one of the key mechanisms that associates with the development and progression of DKD (12). This raises interest in the pathways that regulate insulin sensitivity of podocytes and specifically in the perturbations in these pathways leading to the development of insulin resistance of these specialized cells. Understanding the mechanisms that lead to reduced response of podocytes to insulin and the functional consequences will help to design treatment strategies to prevent the progression of DKD. Several recent, excellent reviews summarize the effects of insulin on podocytes and regulation of renal insulin signaling (13, 14). In this review, we summarize insulin signaling in podocytes in the context of glucose uptake and specifically concentrate in describing the current knowledge on the glucose transporters (GLUTs) and the regulation of GLUT trafficking in podocytes.

## Insulin Signaling Pathways That Regulate GLUT Trafficking

### Insulin Signaling *via* the PI3K/AKT Pathway

The actions of insulin in cells are initiated by binding of insulin to its receptor on the cell surface (Figure 1C). Insulin receptor (IR) is a heterotetrameric complex, consisting of two extracellular α subunits that bind insulin and two transmembrane β subunits with tyrosine kinase activity (15). IR exists in two forms, A and B. IR-A is ubiquitously expressed, and IR-B is expressed in insulin-sensitive tissues such as liver, muscle, adipose tissue, and kidney. Insulin may also bind to and signal *via* insulin-like growth factor I (IGF-IR) or IR/IGF-IR hybrid receptors, although insulin binds to IGF-IR at much lower affinity than to IR [reviewed in Ref. (16)].

Binding of insulin to the α subunit of IR induces transphosphorylation of one β subunit by another on specific tyrosine residues, resulting in increased catalytic activity of the kinase. The receptor further undergoes autophosphorylation at other tyrosine residues. The activated receptor then phosphorylates tyrosine residues on intracellular substrates, including members of the insulin receptor substrate family (IRS1–4) (17). Upon tyrosine phosphorylation, IRS proteins interact with the p85 regulatory subunit of the PI3K, which leads to its activation and translocation to the plasma membrane. PI3K catalyzes phosphorylation of phosphatidylinositol 4,5-bisphosphate to form phosphatidylinositol 3,4,5-trisphosphate (PIP_3_). Insulin-stimulated increase in PIP_3_ results in the phosphorylation and activation of the serine/threonine kinase AKT, also called protein kinase B (PKB), leading to a cascade of signaling events that coordinate trafficking of GLUT4 to the plasma membrane (Figure 1C). In the absence of insulin signal, GLUT4 resides in cytoplasmic vesicular structures. Upon insulin stimulation, GLUT4 is sorted into insulin-responsive vesicles (IRVs) that translocate to the plasma membrane leading to glucose uptake into cells [for reviews on adipocytes and myocytes, see Ref. (18, 19)]. These vesicles are often called GLUT4 storage vesicles or IRVs. However, the various vesicles containing GLUT4 are hard to distinguish from each other (18) and therefore we will call them here generally as GLUT4-containing vesicles (GCVs). Various studies indicate that not only PI3K is essential for GLUT4 translocation and glucose uptake (20) but also other signals, generated by insulin, participate in stimulating translocation of GLUT4 (21–24). IR has also been shown to localize in lipid rafts on the plasma membrane and to induce glucose uptake into cells *via* a PI3K-independent pathway involving Cbl/Cbl-associated protein (25)/TC10 (22). This pathway is involved in insulin signaling and glucose uptake in adipose tissue and muscle cells (22), but apparently the pathway is not active in podocytes (26).

Podocytes express all the elements of the insulin signaling cascade, such as IR, IRS1 (10), and IRS2 (27). Analyses have demonstrated that podocytes have the highest levels of both IR and IRS1 expression when compared with endothelial and mesangial cells in primary culture (28). Similar to adipocytes and muscle cells, podocytes respond to insulin by activating the PI3K and mitogen-activated protein kinase signaling pathways, but only PI3K is implicated in glucose uptake. In podocytes, insulin induces rapid translocation of GLUT4 to the plasma membrane similarly as in muscle cells and adipocytes (10). This induces remodeling of the cortical actin cytoskeleton and contraction (26), allowing podocytes to physiologically respond to the increased glomerular pressure and filtration that happen after a meal.

### Regulation of the PI3K/AKT Pathway in Podocytes

Defects in any site of the insulin signaling pathway, both upstream and downstream of IRS, may arise and disrupt the signaling cascade. Examination of the protein and activity levels of the IR, PI3K, and AKT demonstrate a clear disturbance in these signaling molecules in diabetic conditions (29). Downregulation of IR-B subunit was observed in diabetic podocytes (11). Furthermore, specific deletion of IR in podocytes in mice induces a disease state reminiscent of DKD, without hyperglycemia (3). This demonstrates the necessity of insulin signaling in podocytes for maintaining normal kidney function.

Multiple studies suggest that inhibition of AKT activation is one of the key factors leading to insulin resistance of podocytes. In podocytes of db/db mice, a model for T2DM, insulin-stimulated AKT phosphorylation (activation) is lost (11). A similar loss of insulin signaling *via* PI3K was found in the glomeruli of streptozotocin (STZ) and Zucker rats (28), models for T1DM and T2DM, respectively. The interpodocyte SD with its major component nephrin (30) has emerged as an important signaling center, as nephrin associates with a number of membrane and cytosolic proteins thereby connecting the SD to various signaling pathways (31–33). Concomitantly, nephrin has been shown to activate AKT *via* PI3K (33, 34). Interestingly, nephrin has been found to be downregulated or mislocalized in various models of DKD (35–40).

The negative regulators of the PI3K signaling pathway include lipid phosphatases SHIP2 (SH2-domain-containing inositol polyphosphate-5 phosphatase 2) and phosphatase and tensin homolog (PTEN) (41). These phosphatases dephosphorylate PI(3,4,5)P_3_ to PI(3,4)P_2_ and PI(4,5)P_2_, respectively (42). In line with this, overexpression of SHIP2 downregulates insulin response in cultured human podocytes by reducing AKT activation (43). Furthermore, SHIP2 expression was found to be elevated in glomeruli of insulin resistant obese Zucker rats prior to the rats developed albuminuria (43). Insulin resistance in podocytes due to high glucose could also be a consequence of increased PTEN protein levels, which occurs in an AMP-activated protein kinase (AMPK)-dependent manner (44). Interestingly, lack of *Irs2* renders podocytes insulin resistant due to upregulation of PTEN (27). High glucose also increases the expression of protein tyrosine phosphatase SHP-1, conferring to insulin unresponsiveness of podocytes (45). Studies have also shown that in glomeruli of db/db mice, upregulation of C-jun N-terminal kinase, a negative regulator of insulin signaling, may result in the inability of podocytes to respond to insulin (46).

### Insulin Signals *via* the PKG Pathway in Podocytes

Another signaling pathway that is activated by insulin and stimulates glucose uptake into podocytes is the cGMP-dependent protein kinase G (PKG) pathway (44), which is also involved in glucose uptake into smooth muscle cells (47). cGMP-dependent protein kinase G type I (PKG-I) exists as two isoforms, Iα and Iβ. Dimerization of two PKG-I subunits increases the catalytic activity of PKG-I and consequently enhances its biological action (48). The PKG-Iα isoform is expressed in cultured rat podocytes, in which the activation of PKG-I by insulin or hydrogen peroxide leads to the activation of the insulin signaling pathway *via* increased phosphorylation of IR and AKT. This enhances translocation of GLUT4 to the plasma membrane and increases glucose uptake into cells (Figure 1C) (49). The effect is abolished by a PKG-I inhibitor confirming the role of PKG-I in glucose uptake into podocytes (49).

### Regulation of the PKG Pathway in Podocytes

In podocytes, PKG-Iα is mainly expressed in its monomeric form (50). Insulin increases activation (dimerization) of PKG-Iα in a ROS-dependent manner (50, 51). Insulin may also activate PKG-Iα *via* TRPC6, increasing the permeability of podocyte monolayers to albumin (50). PKG-Iα is upregulated in the glomeruli isolated from obese, hyperinsulinemic, and insulin-resistant Zucker rats compared to lean controls (52).

## Regulation of GLUT Trafficking in Podocytes

### GLUTs and Their Translocation Machinery

Thus far 14 members belonging to the family of facilitative GLUTs have been identified in mammals (53). The facilitative GLUTs, which mediate uptake of glucose into cells down its concentration gradient, are either constitutive or inducible by insulin. Constitutive GLUTs, such as GLUT1, transport glucose into cells at basal condition, and GLUT4 is the major insulin-inducible GLUT. In adipocytes and myocytes in the basal state, GLUT4 is preferentially located in the intracellular vesicular compartments and slowly cycles to the plasma membrane and back. However, upon insulin stimulation, GLUT4 is translocated fast to the plasma membrane in insulin-responsive GCVs [reviewed in Ref. (18, 19)].

GLUT4-containing vesicle trafficking has been studied extensively in adipocytes and myocytes [reviewed in Ref. (18, 19, 54)]. GCV translocation occurs in multiple stages including approaching, tethering, docking, and fusion. The cytoskeleton provides a route for the GCVs to approach the plasma membrane. Depending on the cell type and distance to be trafficked, both actin–myosin and microtubule–kinesin machineries are utilized for GCV translocation (55). An increasing amount of evidence supports a critical role for actin in GLUT4 translocation (10, 55–57). Insulin elicits a rapid, dynamic remodeling of actin filaments into a cortical mesh in various insulin-sensitive cell types, such as differentiated muscle cells, adipocytes, and podocytes (26, 57, 58). Cortical actin is a necessity for GLUT4 translocation and pharmaceutical disruption of cortical actin filament formation inhibits insulin-stimulated GLUT4 translocation (56). The second step includes two processes: tethering and docking. Actin and the exocyst complex proteins help to tether the GCVs to the plasma membrane (18), and docking is mediated by the assembly of the *N*-ethylmaleimide-sensitive factor attachment protein receptor (SNARE) complex (59, 60); reviewed in Ref. (61). The SNARE complex includes a vesicle-SNARE (v-SNARE) on GCVs, vesicle-associated membrane protein 2 (VAMP2), and target-SNAREs (t-SNARE) on the plasma membrane, such as syntaxin 4 and synaptosome-associated protein, 23 kDa (SNAP23) (62, 63). The last step of GCV trafficking to the cell surface is fusion, in which a specific interaction between v-SNARE and t-SNARE proteins allows merging of the lipid bilayers of the GCVs and the plasma membrane [reviewed in Ref. (18, 61)].

### GLUTs Expressed in Podocytes

Podocytes express several GLUTs, including GLUT1 (10, 64), GLUT2 (65), GLUT3 (64), GLUT4 (10, 64–66), and GLUT8 (66).

#### GLUT1

GLUT1 regulates glucose uptake at the basal stage in podocytes, but interestingly, it has also been shown to respond to insulin stimulation in these cells (10). GLUT1 has a vesicular distribution within the cytoplasm and at the plasma membrane, appearing both at apical and basolateral domains of the podocyte foot processes in human glomeruli *ex vivo* (10). The expression of GLUT1 mRNA in the glomeruli of normoalbuminuric T1DM patients was shown to be downregulated, but glomeruli from T1DM patients with microalbuminuria presented increased GLUT1 mRNA expression compared with non-diabetic controls (67). The expression of GLUT1 mRNA and protein followed a similar pattern in the glomeruli of db/db mice (67). Upregulation of GLUT1 was also described in the glomeruli of the STZ-induced rats (68). Studies in cultured cells revealed that GLUT1 expression is elevated in cultured human podocytes exposed to high glucose (69). A similar increase was observed in cultured mesangial cells exposed to high glucose and this associated with increased glucose uptake (70) and stimulated production of extracellular matrix proteins (64). Studies in mice overexpressing GLUT1 in either mesangial cells or podocytes revealed interesting cell type-specific outcomes in terms of DKD. Overexpression of GLUT1 in mesangial cells in mice mimicked typical features of diabetic glomerular disease, without diabetes or hypertension (71). However, podocyte-specific overexpression of GLUT1 in diabetic mice reduced mesangial expansion and fibronectin accumulation, both typical features of DKD (72). This could be mediated by reduced glomerular expression of vascular endothelial growth factor, known to contribute to mesangial matrix accumulation (72). It thus appears that increased expression of GLUT1 in mesangial cells is deleterious whereas increased expression of GLUT1 in podocytes protects against DKD. A protective role for GLUT1 is supported by the finding that peroxisome proliferator-activated receptor (PPAR) γ agonist rosiglitazone, shown to prevent kidney disease in a mouse model of T1DM (73) and to reduce albuminuria in patients with T2DM (74), increases glucose uptake into podocytes by enhancing membrane localization of GLUT1 (75).

#### GLUT4

GLUT4 has a cytoplasmic, vesicular distribution in the resting cell, but upon insulin stimulation, GLUT4 translocates to the cell surface [reviewed in Ref. (18, 19)]. In human glomeruli *ex vivo*, GLUT4 localizes in an intracellular vesicular distribution and at the apical and basolateral domains of the plasma membrane of the podocyte foot processes (10). Contrary to GLUT1, the expression of GLUT4 mRNA was shown to be upregulated in the glomeruli of normoalbuminuric T1DM patients (67), whereas glomeruli from T1DM patients with microalbuminuria presented decreased GLUT4 mRNA expression compared to non-diabetic controls (67). GLUT1 mRNA and protein expression followed a similar pattern in db/db mice (67). Also, contrary to GLUT1, chronic exposure of cultured human podocytes to high glucose reduced GLUT4 expression (69). Interestingly, podocyte-specific GLUT4-deficient mice do not develop albuminuria even though they have fewer and larger podocytes than the wild-type mice (67). Furthermore, they are protected from diabetes-induced podocyte hypertrophy, mesangial expansion, and albuminuria (67). The mice showed increased activation of AMPK in glomeruli and suppression of the mammalian target of rapamycin (mTOR) pathway (67), proposing that lack of GLUT4 affects nutrient sensing in podocytes. Both clinical and experimental data support a role for nutrient sensing signals (mTORC1, AMPK) in the pathogenesis of the kidney complication in diabetes (76). AMPK activity has been shown to be decreased in the kidneys of several types of diabetic rodent models, including STZ-induced type 1 diabetic rats (77, 78) and type 2 diabetic db/db mice (79, 80), leading to renal hypertrophy or renal interstitial fibrosis. Hyperactivation of the mTORC1 signal is strongly associated with the progression of podocyte injury and proteinuria in diabetic animal models, characterized by dysregulation of nephrin and podocyte loss (39, 81). These data suggest that high GLUT4 level would be an enemy in DKD and that decreasing GLUT4 expression or attenuating its function may be beneficial in diabetic kidney. Additional work is needed to investigate whether the function of GLUT4 in podocyte is independent of insulin signaling, as a study suggests that GLUT4 may directly regulate actin remodeling (82).

#### Other GLUTs

GLUT2 has been described to mediate glucose uptake in cultured rat podocytes (65). GLUT3 expression has been shown to be upregulated in human podocytes exposed to high glucose *in vitro* (69), and GLUT8 expression was higher in podocytes of kidneys of diabetic db/db mice compared with non-diabetic mice (66). Defining the roles of these GLUTs in podocyte function awaits further studies in rodent models of diabetes and DKD.

#### Properties of GLUTs and Metabolism of Podocytes

GLUT1, GLUT3, GLUT4, and GLUT8 are high-affinity, low-capacity GLUTs, and GLUT2 is a low-affinity, high-capacity GLUT (83). As individual transporters may get saturated, the presence of the high-affinity GLUTs at the plasma membrane plays an important role in regulating the flux of glucose into cells. As described earlier, the studies thus far have defined the expression of GLUT1 and GLUT4 only at the mRNA level in glomeruli of patients with diabetes (67), and the study defining their localization by immunoelectron microscopy in human podocytes concentrated looking at only normal human kidney tissue (10). It would be interesting to define the exact subcellular localization of each GLUT in podocytes during the progression of DKD, but this is challenging due to the invasiveness of obtaining tissue material. For the same reason, regulation of the metabolism of podocytes in diabetic conditions has mainly relied on studies carried out using cultured human podocytes treated with factors associated with diabetes. In line with this, during differentiation in normal (5 mM) glucose, cultured human podocytes activate oxidative metabolism and reduce glycolytic enzymes (84). Hyperglycemic conditions (20 mM glucose) promote metabolic reprogramming in podocytes, with reduction of mitochondrial biogenesis and increased glycolysis (84). Corresponding signs of glycolytic switch are observed in kidney sections of human patients with DKD (84). This is consistent with a previous study showing that mitochondrial function is dysregulated in patients with DKD (85).

### Molecular Regulators of GLUT Trafficking in Podocytes

**Figure 2:**
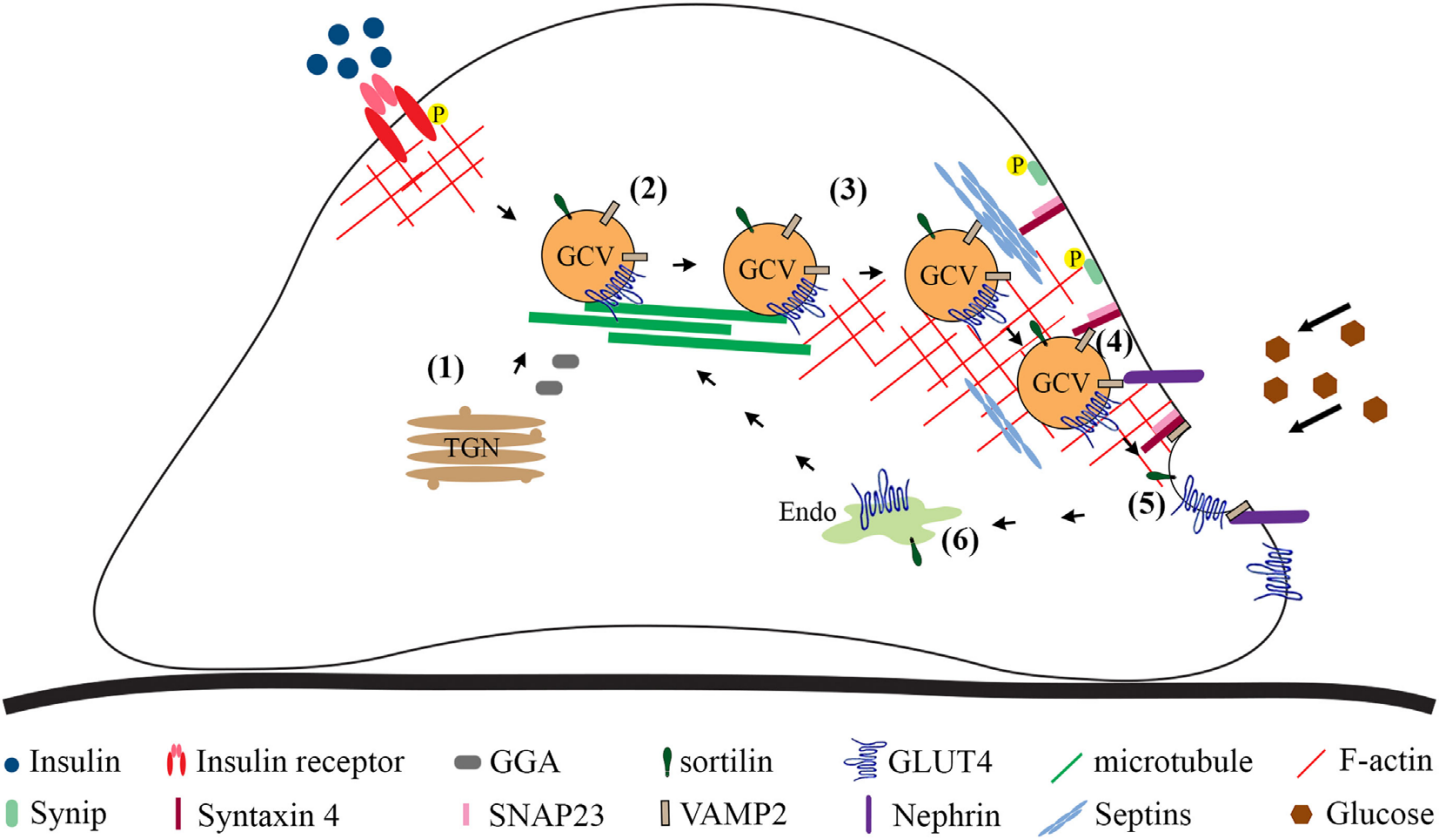
Overview of the steps involved in GLUT4 trafficking to the plasma membrane based on studies carried out in cultured podocytes. Newly synthesized GLUT4 molecules are sorted directly into GCVs (1). GLUT4 may be sorted into GCVs also from other vesicular compartments. Cytoskeleton plays a role in approaching of the vesicles from the perinuclear region to the plasma membrane (2). Tethering (3), docking (4), and fusion (5) are required to merge the lipid bilayer of the GCV with the plasma membrane. Once at the plasma membrane, the GCV docking (4) and fusion (5) require formation of the ternary complex between v-SNARE, VAMP2, on the GCV and t-SNAREs, syntaxin-4 and SNAP23, on the plasma membrane, allowing the extracellular exposure of the GLUT4. (6) GLUT4 present at the plasma membrane is endocytosed and transported to the endosomal system for recycling. GCV, GLUT4-containing vesicle; P, phosphorylation; Endo, endosomes and recycling endosomes; TGN, trans-Golgi network; SNAP23, synaptosome-associated protein, 23 kDa; SNARE, *N*-ethylmaleimide-sensitive factor attachment protein receptor; t-SNARE, target SNARE; v-SNARE, vesicle SNARE; VAMP2, vesicle-associated membrane protein 2.

In the myoblasts and adipocytes in the basal state, at least half of the GLUT4 population is found in a vesicle compartment. Stimulation with insulin increases the amount of GLUT4 at the cell surface mainly by promoting exocytosis of GCVs and to a lesser extent by increasing exocytosis from the recycling system, orchestrated by an array of regulatory and sorting proteins (10, 86, 87). In addition, glucose uptake into cells may be regulated by affecting the endocytosis of GLUT4 at the plasma membrane. Mechanisms that regulate GCV trafficking and glucose uptake into podocytes are less well defined than in adipocytes and myocytes, and currently there are no data describing the regulation of GLUT4 endocytosis in podocytes. Here, we summarize the current knowledge on the mechanisms by which sorting of GLUT4 into GCVs and insulin-stimulated GCV exocytosis are regulated in podocytes (Figure 2).

#### Sorting

Cultured podocytes depleted of CD2-associated protein show attenuated glucose uptake in the basal state compared with wild-type podocytes, and CD2AP knockout podocytes fail to increase glucose uptake in response to insulin (88). This process was found to be independent of the insulin signaling pathway (as measured by insulin-induced AKT activation) or altered expression of GLUT1 and GLUT4, proposing a defect in GLUT4 trafficking. In line with this, GLUT4 appears as clusters in the perinuclear region of podocytes lacking CD2AP (88). GLUT4 in the perinuclear region is sorted into GCVs prior to transport toward the plasma membrane (Figure 2). This step is controlled by GGA2, which facilitates the formation of GCVs by recruiting clathrin and adaptor proteins, leading to enhanced insulin-stimulated glucose uptake (89, 90). Of note, podocytes are the only cells in the kidney that express GGA2 (91). Interestingly, CD2AP forms a complex with GGA2, and the complex formation is further increased by insulin stimulation, proposing that CD2AP regulates GCV sorting.

GGA2 binds a transmembrane protein, sortilin, a key component in the formation of GCVs (92). Sortilin overexpression stabilizes GLUT4 (92), and its upregulation in CD2AP-depleted podocytes could explain the increased protein level of GLUT4 (88). Sortilin overexpression has been shown to increase the efficiency of the formation of GCVs and to induce glucose uptake into adipocytes (92) and myocytes (93). Despite upregulation of GLUT4 and sortilin in the absence of CD2AP, GLUT4 was not efficiently trafficked to the plasma membrane in response to insulin (88). This suggests that increased expression of endogenous GLUT4 and sortilin in CD2AP knockout podocytes is a compensatory mechanism, possibly caused by a defect in GCV formation or trafficking. Interestingly, CD2AP forms a complex also with clathrin and, apparently *via* its ability to directly bind actin (94), connects clathrin to actin in the perinuclear region (88). When CD2AP is absent, the recycling of clathrin back to the trans-Golgi membranes from the vesicular fraction containing GCVs appears impaired. This reduces insulin-stimulated trafficking of GCVs resulting in reduced glucose uptake when CD2AP is absent (88).

#### Approaching and Tethering

The precise mechanism by which GCVs are delivered to the plasma membrane in podocytes is not well defined, but it has been shown that the translocation depends on an intact actin cytoskeleton (10, 26). Insulin-treated podocytes display cortical reorganization of actin, which occurs *via* activation of RhoA and inhibition of CDC42 (10, 26). Also ezrin, an actin-binding protein, induces dynamic remodeling of actin in podocytes and the process involves the actin-severing protein cofilin-1 (95). Loss of ezrin in cultured podocytes increases glucose uptake, but apparently this does not occur due to enhanced trafficking of GLUT4. In response to insulin, podocytes absorb glucose not only *via* GLUT4 but also *via* the constitutive GLUT, GLUT1 (10). In ezrin-depleted podocytes, GLUT1 was observed at the plasma membrane in basal, starved, and insulin-stimulated conditions, proposing that an increase in glucose uptake in ezrin-deficient podocytes is due to enhanced trafficking of GLUT1 to the plasma membrane (95). Interestingly, ezrin is downregulated in the glomeruli of obese Zucker rats and in podocytes of human patients with T2DM without clinical nephropathy or histopathological diagnostic signs of DN (95). The effect of ezrin downregulation on GLUTs and glucose uptake in the glomeruli *in vivo* awaits further studies.

#### Docking and Fusion

The final step of the GCV trafficking requires docking and fusion machinery that merges the lipid bilayer of the GCV with that of the plasma membrane. The SD protein nephrin plays an important role in this process, as podocytes deficient in nephrin or with missense mutations in nephrin are insensitive to insulin with respect to glucose uptake (96). Nephrin interacts with several key regulators of GLUT4 trafficking, including the vesicle-associated VAMP2 on GCVs, and facilitates the insulin-stimulated GCV fusion with the plasma membrane (96). Nephrin also forms a complex with the small filamentous GTPase septin 7, which negatively regulates glucose uptake into podocytes (97). Knockdown of septin 7 strengthens the interaction between nephrin and VAMP2 and also between syntaxin 4 and VAMP2 (97) (Figure 2). This, supported by the previous models proposed for septin-5/CDCrel-1 (98), corroborates the idea that septin 7 forms a physical barrier that hinders GCV trafficking. Thereby, depletion of septin 7 allows the SNARE complex formation between the v-SNAREs and the plasma membrane-SNAREs (97) (Figure 2). Nucleobindin-2 was recently shown to associate with septin 7 (99). As knockdown of nucleobindin-2 prevents insulin-stimulated translocation of GLUT4 to the plasma membrane, the authors suggested that nucleobindin-2 may reverse septin 7-induced inhibition of insulin-stimulated GLUT4 translocation in podocytes (99). This, however, requires detailed studies to define whether nucleobinding-2 functionally affects septin 7 and to unravel the molecular mechanisms involved.

It is plausible to assume that certain proteins that regulate GLUT4 trafficking in muscle and adipose cells function similarly in podocytes. One such protein is syntaxin 4-interacting protein (synip), which regulates the docking and fusion of GCVs with the plasma membrane in adipocytes (100). Synip occupies the same binding domain on syntaxin 4 that also interacts with VAMP2. Insulin induces phosphorylation of synip on S99, which leads to dissociation of synip from syntaxin 4, thereby vacating the binding site for VAMP2 and allowing the fusion to occur (100) (Figure 2). In line with this, GCVs in podocytes expressing a phosphorylation-deficient Synip mutant (S99A) fail to dock and fuse with the plasma membrane and the cells present with reduced glucose uptake (101).

Another protein that has been shown to regulate glucose uptake in both adipocytes and podocytes is non-muscle myosin IIA (NM-IIA) (102–104). Knockdown of non-muscle myosin heavy chain IIA (NMHC-IIA), a component of the NM-IIA hexameric complex, decreases insulin-stimulated glucose uptake into podocytes (104). Insulin stimulation activates NM-IIA by phosphorylating the regulatory light chain subunit of the complex, and this enhances GCV docking and fusion with the plasma membrane (103). Interestingly, nephrin, septin 7, and the plasma membrane SNARE protein SNAP23 form a complex with NMHC-IIA (104). Septin 7 is a regulator of the NM-IIA activity in the SNAP23 complex, as knockdown of septin 7 enhances the phosphorylation of the NM-IIA regulatory light chain in the SNAP23 complex. In line with this, insulin stimulation is coupled with a decrease in septin 7 level and an increase in the activity of NM-IIA in the SNAP23 complex, enhancing GCV docking and fusion and increasing glucose uptake into podocytes (104). Thus, in addition to forming a physical barrier, septin 7 reduces glucose uptake into podocytes by reducing the activity of NM-IIA in the plasma membrane SNARE complex. In diabetic rat glomeruli and cultured human podocytes exposed to macroalbuminuric sera from patients with T1DM, the activity of NM-IIA is increased (104), potentially leading to an increase in glucose uptake.

## Insulin Resistance Associates with the Development of DKD

### Mechanisms Leading to Insulin Resistance

Insulin resistance is a condition in which cells fail to respond to the normal actions of insulin. At the cellular level, the mechanisms leading to the development of insulin resistance may include mutations in the IR itself, impairments in the PI3K/AKT signaling pathway or perturbations in the GLUT trafficking. These changes may lead to reduced uptake of glucose into cells and contribute to the development of hyperglycemia.

Several pieces of evidence suggest that reduced action of insulin may play a role in the development of DKD. Insulin resistance has been reported to correlate with microalbuminuria in patients with T1DM (4) or T2DM (5–7) and also in non-diabetic subjects (8). Both clinical and experimental data suggest that insulin sensitizers have a renoprotective role in patients with diabetes (105) as well as in experimental animal models of diabetes (73, 106, 107). Interestingly, podocytes are insulin sensitive and share similarities with skeletal muscle cells and adipocytes in respect to the kinetics of the insulin-stimulated glucose uptake and the expression of GLUTs, including GLUT1 and GLUT4 (10). Due to the invasiveness, it is challenging to define whether podocytes in human patients with diabetes develop insulin resistance and to determine whether podocyte insulin resistance *per se* contributes to the development of DKD. Identification of normoglycemic, insulin-resistant patients presenting DKD support the role of insulin resistance as a contributing factor in the pathogenesis of DKD, as described in a recent case report (108). However, these cases are apparently few, suggesting that insulin resistance alone is not enough to lead to the development of DKD. Also, only some of the patients with mutations in IR and severe insulin resistance develop DKD, and some develop other kidney diseases (109), suggesting that insulin resistance, in combination with other factors, may contribute to kidney injury. A recent review summarizes the consequences of insulin resistance and the potential mechanisms associated with the progression of DKD (12). These data highlight the importance of understanding the mechanisms by which insulin sensitivity of podocytes is regulated and helps to identify new targets and define new treatment strategies for kidney diseases involving insulin resistance.

## Factors Regulating GLUTs and their Trafficking in Podocytes

**Figure 3:**
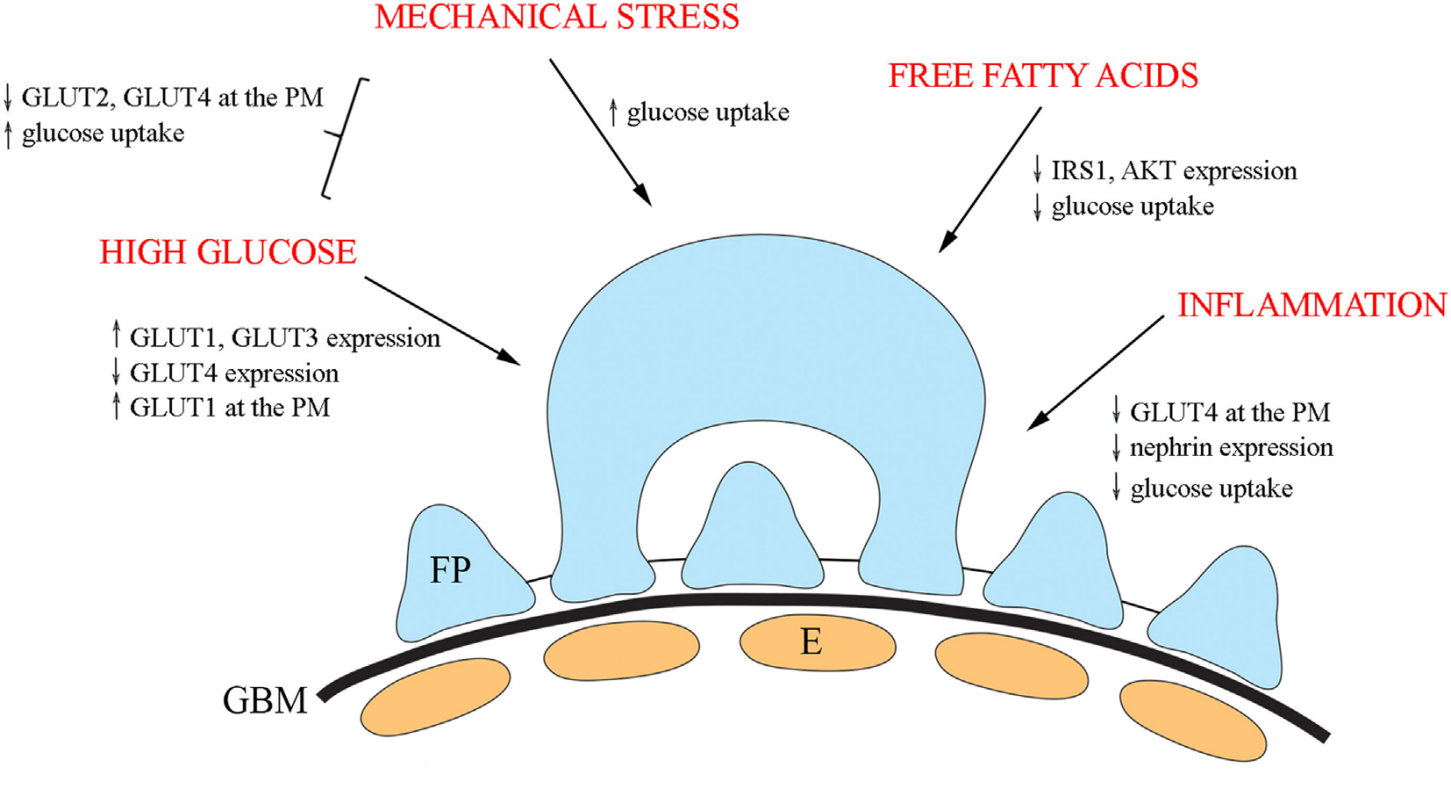
An overview of the diabetes-associated external factors regulating glucose transporters and glucose uptake in podocytes. In cultured podocytes, high glucose leads to upregulation of GLUT1 and GLUT3 and downregulation of GLUT4 and increases the presence of GLUT1 at the plasma membrane. Mechanical stress increases glucose uptake and in combination with high glucose, decreases the expression of both GLUT2 and GLUT4 at the plasma membrane, and increases glucose uptake. Free fatty acids lead to reduced phosphorylation of IRS1 and AKT and diminished glucose uptake. Also, inflammatory mechanisms impair insulin signaling and insulin-induced glucose uptake in cultured podocytes. E, endothelial cell; FP, foot process; GBM, glomerular basement membrane.

Insulin signaling in podocytes is influenced by various factors and has been reviewed elsewhere (13, 110). Here, we shortly summarize diabetes-associated external factors shown to influence GLUTs and their trafficking (Figure 3).

In diabetes, podocytes are exposed to high glucose concentrations prompting to carry out analyses on the effect of high glucose on the expression of GLUTs. High glucose treatment of cultured human podocytes led to the upregulation of GLUT1 and GLUT3 and downregulation of GLUT4 (69). In addition, high glucose increased the presence of GLUT1, but not of GLUT3 or GLUT4, at the plasma membrane (69). Another study defined the effect of mechanical stress, modulating increased intracapillary pressure observed in diabetes, in combination with high glucose on glucose uptake using cultured rat podocytes (111). The study revealed that mechanical stress increased glucose uptake, and the effect was potentiated by high glucose. The combination of high glucose and mechanical stress decreased the expression of both GLUT2 and GLUT4 at the plasma membrane, suggesting that the increase in glucose uptake is mediated by other GLUTs under these conditions (111).

Of obesity and insulin resistance-associated factors, free fatty acids play a central role in the progression of T2DM. Treatment of cultured human podocytes with palmitate, the predominant circulating saturated free fatty acid, leads to reduced phosphorylation of IRS1 and AKT and diminished glucose uptake (112). An increasing number of studies indicate that activation of the innate immune system, and inflammatory mechanisms are important in the pathogenesis of DKD (113–117). Nucleotide-binding oligomerization domain containing 2 (NOD2), a member of the NOD-like receptor family, plays an important role in innate immune response and has been shown to be upregulated in the kidney in an experimental model and patients with diabetes (118). In line with this, depletion of NOD2 was found to protect against diabetes-induced kidney injury. NOD2 was further observed to impair insulin signaling and insulin-induced glucose uptake in cultured podocytes by inhibiting GLUT4 translocation to the plasma membrane (118). Knockdown of NOD2 expression also attenuated nephrin downregulation induced by high glucose. This is of importance in the context that nephrin enhances GCV docking and fusion with the plasma membrane (96). Interestingly, also activated macrophages downregulate nephrin expression *via* TNFα and induce podocyte injury (119).

## Conclusion

Diabetic kidney disease is the leading cause of end-stage renal disease worldwide. Treatments targeting hyperglycemia and blood pressure combined with lifestyle interventions have not been able to stop the progression of this devastating disease. This calls for continued research on the pathophysiological mechanisms associated with the progression of DKD, aiming to identify new targets for drug development. Studies have shown that insulin-sensitizing agents, including metformin and PPARγ agonists, are beneficial in preventing kidney damage in both T1DM (120) and T2DM (121) as well as in non-DKD (122). PPARγ agonist rosiglitazone enhances glucose uptake into podocytes by enhancing GLUT1 translocation to the plasma membrane (75), and remarkably, GLUT1 overexpression in podocytes protects against DKD (72). Concomitantly, proteins that enhance the presence of GLUT1 at the plasma membrane could have therapeutic potential in preventing the development and progression of DKD.

Interestingly, research has revealed that molecules associated with insulin signaling and glucose uptake in podocytes have importance in a context wider than just glucose uptake. Accordingly, GLUT4 plays a role in podocyte nutrient sensing, and interestingly, depletion of GLUT4 protects podocytes from DKD by reducing mTOR signaling (67). In addition to mTOR, nutrient-sensing signals AMPK and Sirt1 are altered in the diabetic kidney [reviewed in Ref. (76)]. Autophagic activity, which is regulated by the above-mentioned nutrient-sensing signals, is also altered in both podocytes and proximal tubular cells under diabetic conditions (123, 124). This proposes that molecules that reduce the expression or functional activity of GLUT4 or affect the nutrient sensing pathways in podocytes could provide potential treatment targets in DKD. In the next few years, additional studies addressing these pathways as well as defining the functions of other, thus far less studied GLUTs, GLUT2, GLUT3, and GLUT8, in podocytes and potentially elsewhere in the nephron, may ultimately provide a clearer perspective for the development of new drugs. The new roles may include, in addition to the expected functions in glucose uptake, novel functional indications and pathways independent of insulin signaling and glucose uptake. Finally, it will be important to define the functional interplay between the various pathways to elucidate the regulatory networks that maintain the normal function of podocytes in health or when disturbed and lead to podocyte dysfunction and development of albuminuria.

## Author Contributions

AW and SL contributed to conception and design, performed literature searches, and wrote the manuscript.

## Conflict of Interest Statement

The authors declare that the research was conducted in the absence of any commercial or financial relationships that could be construed as a potential conflict of interest.
